# Chemical-Specific T Cell Tests Aim to Bridge a Gap in Skin Sensitization Evaluation

**DOI:** 10.3390/toxics12110802

**Published:** 2024-11-06

**Authors:** Nele Fritsch, Marina Aparicio-Soto, Caterina Curato, Franziska Riedel, Hermann-Josef Thierse, Andreas Luch, Katherina Siewert

**Affiliations:** 1 German Federal Institute for Risk Assessment (BfR), Department of Chemical and Product Safety, Dermatotoxicology Study Centre, 10589 Berlin, Germany; nele.fritsch@bfr.bund.de (N.F.); caterina.curato@bfr.bund.de (C.C.); franziska.riedel@bfr.bund.de (F.R.);; 2 Institute of Biotechnology, Technical University of Berlin, 10115 Berlin, Germany; 3 Institute of Pharmacy, Freie Universität Berlin, 14195 Berlin, Germany

**Keywords:** adverse outcome pathway, allergic contact dermatitis, chemical-induced T cell epitopes, contact allergens, in vitro T cell test, key events, new approach methodologies, skin sensitization, T cell receptor

## Abstract

T cell activation is the final key event (KE4) in the adverse outcome pathway (AOP) of skin sensitization. However, validated new approach methodologies (NAMs) for evaluating this step are missing. Accordingly, chemicals that activate an unusually high frequency of T cells, as does the most prevalent metal allergen nickel, are not yet identified in a regulatory context. T cell reactivity to chemical sensitizers might be especially relevant in real-life scenarios, where skin injury, co-exposure to irritants in chemical mixtures, or infections may trigger the heterologous innate immune stimulation necessary to induce adaptive T cell responses. Additionally, cross-reactivity, which underlies cross-allergies, can only be assessed by T cell tests. To date, several experimental T cell tests are available that use primary naïve and memory CD4+ and CD8+ T cells from human blood. These include priming and lymphocyte proliferation tests and, most recently, activation-induced marker (AIM) assays. All approaches are challenged by chemical-mediated toxicity, inefficient or unknown generation of T cell epitopes, and a low throughput. Here, we summarize solutions and strategies to confirm in vitro T cell signals. Broader application and standardization are necessary to possibly define chemical applicability domains and to strengthen the role of T cell tests in regulatory risk assessment.

## 1. Introduction

In our modern industrialized society, humans are regularly exposed to numerous chemical compounds. Single or repeated skin contact with sensitizing chemicals may induce contact allergies in susceptible individuals. Upon re-exposure, an inflammatory skin condition known as allergic contact dermatitis (ACD) can develop [1,2,3]. Epidemic-like occurrence of contact allergies has been reported in recent decades [4,5]. Rough estimations claim that approximately 20% of the general population may be sensitized to at least one contact allergen [6]. The widespread occurrence of ACD implies significant economic and personal costs [7].

To prevent or reduce ACD, newly authorized chemicals must undergo skin sensitization testing to comply with national and international regulations on chemical safety. For instance, in the European Union (EU), for all chemicals manufactured or imported in quantities of ≥1 ton per year, information on skin sensitizing properties is required by the REACH regulation (Registration, Evaluation, Authorization and Restriction of Chemicals; Regulation (EC) No. 1907/2006).

**Figure 1:**
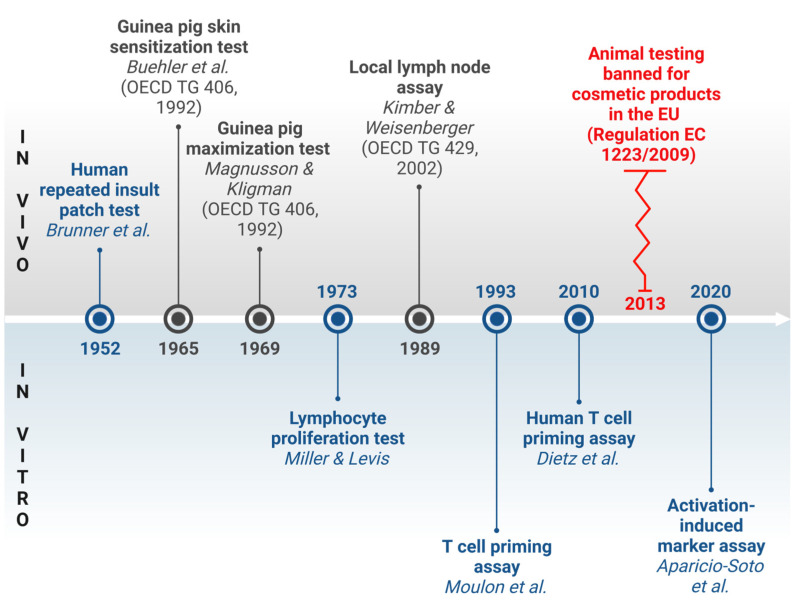
History of tests for assessing chemical-induced T cell responses. Traditional in vivo methods for assessing T cell responses in animals are represented in the OECD test guidelines (upper part) [8,9,10,12]. For a long time, these methods preceded the development of in vitro assays (lower part). Nevertheless, T cell assays have significantly advanced over time [16,17,18,19]. The necessity of alternative tests was demonstrated by the prohibition of animal testing for cosmetics in the EU in 2013 (red zigzag line). References were selected from among the earliest descriptions of these tests for studying contact allergens. The terms ‘assay’ and ‘test’ are used interchangeably. Blue text indicates human-based test systems, while dark gray denotes animal-based methods. EC—European Commission; EU—European Union; OECD—Organization for Economic Co-operation and Development; TG—test guideline. Created in BioRender.com. Siewert, K. (2024) https://BioRender.com/h57s252, accessed on 1 November 2024.

For decades, animal-based tests have been the gold standard to assess the skin sensitizing properties of chemicals (Figure 1). Guinea pig tests, which address both the sensitization and elicitation phase of ACD, have largely been replaced by the more animal-friendly murine local lymph node assay (LLNA) that only considers the sensitization phase (OECD TG 406; OECD 429) [8,9,10]. Early human patch tests designed to induce active sensitization have been banned under REACH [11,12]. However, human repeated insult patch tests (HRIPTs) are still used to confirm no-effect levels, mainly in the cosmetics, household products, and pharmaceutical sectors [13,14,15].

There is a growing need for alternative methods to replace or reduce in vivo testing, including skin sensitization assessment. In recent years, the concept of new approach methodologies (NAMs) has emerged to accommodate a higher throughput, reduce uncertainties resulting from interspecies differences, and provide insights into molecular mechanisms, while adhering to improved ethical standards [20,21,22]. Cutting-edge NAMs for skin sensitization testing have been incorporated into REACH and other chemical regulations, thereby allowing animal testing only when these alternative methods prove inconclusive [23,24]. The EU’s implementation of a comprehensive ban on animal testing for cosmetics in 2013 underscores the ongoing evolution in the field of chemical regulation (Regulation (EC) No. 1223/2009).

**Figure 2:**
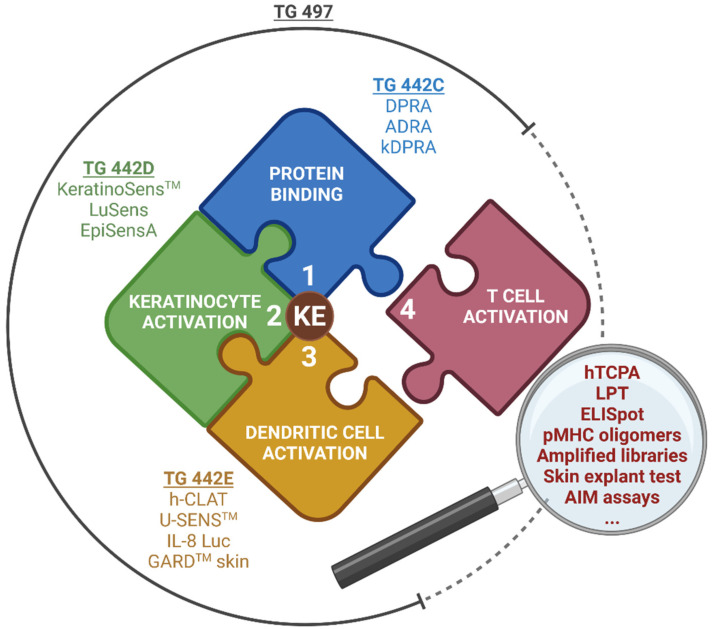
New approach methodologies (NAMs) address different key events (KEs) of the adverse outcome pathway (AOP) of human skin sensitization. Currently, NAMs are available for KE1 to 3, which are also covered by OECD guidelines. KE4 describes T cell activation as the final step of the AOP, but validated methods are still missing. This review describes approaches for measuring chemical-induced T cell responses, which may be further developed into a regulatory accepted predictive KE4 test method. ADRA—amino acid derivative reactivity assay; AIM—activation-induced marker; DPRA—direct peptide reactivity assay; ELISpot—enzyme-linked immunosorbent spot; EpiSensA—epidermal sensitization assay; GARD^TM^ skin—genomic allergen rapid detection for assessment of skin sensitizers; h-CLAT—human cell line activation test; hTCPA—human T cell priming assay; IL-8 Luc—interleukin 8 reporter gene assay; kDPRA—kinetic direct peptide reactivity assay; LPT—lymphocyte proliferation test; pMHC oligomers—peptide-major histocompatibility complex class I and II oligomers; U-SENS^TM^—myeloid U937 skin sensitization test. Created in BioRender.com. Siewert, K. (2024). https://BioRender.com/a64x125, accessed on 1 November 2024.

NAMs for skin sensitization have been developed for a series of biological key events (KEs) considered essential to define the adverse outcome pathway (AOP) of skin sensitization [25] (Figure 2). The molecular initiating event (MIE) covers the binding of chemicals to endogenous skin proteins (KE1, haptenation) and can be addressed by in chemico peptide reactivity assays (OECD TG 442C). Subsequently, keratinocytes and dendritic cells (DCs) become activated (KE2 and 3, respectively), which can be analyzed by cellular in vitro tests (OECD TGs 442D and 442E). Activated DCs are then thought to migrate to the draining lymph nodes, where they present chemical-induced epitopes on their cell surface to prime naïve T cells (KE4). Subsequently, antigen-specific proliferation and differentiation into effector and memory T cells is induced, which terminates the sensitization phase [2,25,26]. For the final KE4, experimental assays are available, but have not yet been validated or accepted for regulatory use (Figure 1 and Figure 2).

So far, no individual NAM can replace in vivo testing for skin sensitization. Still, NAM combinations for KE1 to 3 that also include in silico tools have been implemented successfully as defined approaches (DAs) in OECD TG 497 and the related guidance document (GD 256). These DAs can replace animal testing and allow classification according to the globally harmonized system (GHS) of classification and labelling of chemicals within their applicability domains. Further research to improve the available methods is ongoing [27,28,29]. In this context, a validated T cell test is needed to fill a critical gap by resolving discrepancies among KE1 to KE3 NAMs, LLNA, human HRIPT, and clinical data, as well as to assess cross-reactivities. Still, T cell-mediated adaptive immunity exhibits unique characteristics including clonal and phenotypic diversity, as well as special antigen recognition mechanisms, as outlined in Section 2. These factors have influenced and delayed T cell test development.

In this review, we highlight key challenges due to the complexity of the underlying biology and provide potential solutions to the technical difficulties involved in assay development, expanding on previous work in this field [26,30,31,32].

## 2. Biological Basis of a Regulatory T Cell Test

A predictive T cell test for skin sensitizing properties is based on the assumption that chemical-specific T cells are also present in non-sensitized individuals, even without previous exposure. This phenomenon arises from the vast diversity of T cell receptors (TCRs) and their polyspecificity in humans. Each T cell expresses a unique TCR consisting of an α- and β-chain (or, more rarely, a γ- and δ-chain). The chains are generated in the thymus through recombination of V(D)J gene segments, and random insertions and deletions in the complementary determining region 3 (CDR3). These processes result in a vast theoretical variability of ~10^15^–10^61^ possible TCRs [33,34]. From these theoretical numbers, a huge and mainly unique TCR repertoire is realized in each person, estimated to be about 100 million (10^8^) TCRs [35].

TCR polyspecificity is also referred to as cross-reactivity. This property describes the ability of a single TCR to react to multiple epitopes [36,37,38]. Consequently, the human TCR repertoire covers virtually any epitope, but specific T cells for a certain allergen are only available at low levels in the naïve T cell pool due to the high diversity (~1 specific among 5∙10^4^ to 10^7^ non-specific T cells) [39,40]. A naïve T cell with a given TCR (a clonotype) is represented by a low number of daughter cells (e.g., ~50 to 200 for murine CD8+ T cells) that are circulating between the blood and lymphatic system [41]. Therefore, cells from one naïve T cell clonotype are usually not detectable in several wells of an in vitro culture, a phenomenon known as a sampling effect. In contrast, the effector and memory T cell pools contain a broad range of individual clonotype frequencies, including some that are highly expanded [42,43].

Epitopes recognized by TCRs consist of a peptide presented by a protein of the highly polymorphic major histocompatibility complex (MHC), also known as human leukocyte antigen (HLA) [44]. For most chemical-induced epitopes, the identity of the HLA allele, the presented self-peptide, and the exact haptenation site and mechanism remain unknown [45,46,47,48]. Covalent chemical binding via electrophile/nucleophile or radical mechanisms (haptenation) as well as complex formation (i.e., chelation, also termed pharmacological interaction, or p-i concept) are possible (see also Section 4.1) [3,49,50].

So far, reliable in vitro T cell signals have been observed for only a limited number of contact allergens and drugs [45,47]. A prominent example is nickel (Ni^2+^ ions), which astoundingly activates approximately ~1 in 1000 CD4+ T cells (at ~200 µM), due to the interaction with certain common TCR antigen binding sites [19]. Similar observations were published for cobalt (Co^2+^) and palladium (Pd^2+^) [51]. In an analogous manner, the experimental contact allergen 2,4,6-trinitrobenzene-1-sulfonic acid (TNBS) has been shown to activate many CD4+ and CD8+ T cells [52,53]. Here, the chemical moiety (trinitrophenyl group) dominates the TCR interactions at specific positions, while the sequence of the underlying MHC-presented peptides can vary. This mechanism allows the involvement of many TCRs, possibly with distinct CDR3 motifs [52,54]. While TCR repertoires are largely unique among individuals, bias not only in thymic selection but also among reactive TCRs exists. This encompasses the selection of common gene segments, amino acid motifs in the CDR3, or a combination thereof [34,51,55,56,57,58].

A T cell test is indicated to determine the extent to which a chemical sensitizer activates a broader range of T cells, as well as to distinguish signals from heterologous immune stimulation. Sufficient stimulation of the innate immune system, required for an adaptive T cell response, is thought to be mediated by the reactivity of the chemical and addressed by KE1 to 3 methods [59]. In real-life scenarios, heterologous stimulation from skin injury, barrier defects, infections, or co-exposure to other irritants in mixtures may contribute to innate immune activation [60,61,62]. A prominent example is nickel, predicted to exhibit a weak to moderate potency in available tests and displaying also some irritant characteristics in vitro [63,64,65]. However, nickel allergy is strongly associated with skin injury which is exceptionally immunogenic and could also provide the required heterologous stimulation. Thus, broad T cell reactivity, combined with skin injury, may contribute to the high prevalence of nickel allergy [66,67]. Heterologous immune stimulation is currently not considered in any predictive testing. All reference data on skin sensitizing properties are derived from human or animal models with an intact skin barrier, without the presence of additional chemicals (mixtures) or pathogens, with the exception of sodium dodecyl sulfate (SDS), which was used sometimes in the past. Taken together, the contribution of heterologous immune stimulation to skin sensitization under real-life scenarios remains unclear. However, T cell tests may help to dissect the contributions of innate and adaptive immune activation to skin sensitization for a given sensitizing chemical.

## 3. Available T Cell-Based Assays

Human T cell tests use blood as a readily accessible source of primary T cells and APCs [45]. All assays rely on autologous systems, where T cells and APCs derive from the same donor to prevent reactions against polymorphic HLA proteins (mixed lymphocyte reactions). This setting ensures that the detection of chemical-specific responses will not be hindered by allogeneic stimulation. After antigen exposure, different readouts can be used and the results are compared to cells treated with an unrelated chemical (specificity control) and/or untreated cells (background control). The available tests can be categorized based on their capacity to interrogate naïve, memory, or both T cell populations (Table 1).

### 3.1. Detection of Naïve T Cells

Unlike memory T cells, naïve T cells require further signals beyond TCR engagement, including co-stimulation via CD28 and cytokines like interleukin (IL)-2, for full activation and proliferation [86,87,88]. Professional APCs like DCs supply essential co-stimulatory molecules like CD86 or cytokines, which also drive the shift of T cells towards effector and memory phenotypes including Th1, Th2, or Th17 subpopulations [89].

Priming assays, among the first tests developed for skin sensitizers, are currently the only ones that mimic the sensitization phase in vitro. These assays involve the co-culture of naïve human T cells with APCs in the presence of a chemical, integrating the necessary co-stimulatory signals from the innate immune system [31,90]. This approach not only activates naïve T cells but also potentially allows for the assessment of chemical-induced shifts in immune responses [17]. After the initial priming, cells are restimulated at one or several time points.

T cell responses are primarily evaluated by cell proliferation, traditionally using [^3^H]-thymidine incorporation into DNA [17,18,70]. Results are expressed as counts per minute (cpm) and typically converted into a stimulation index. The stimulation index quantifies cell proliferation by comparing chemical-treated cells to unstimulated control cells under identical conditions, thus accounting for biological variations [69,70]. Alternative techniques have been established to replace radioactive thymidine due to safety concerns and limitations in single-cell resolution. The use of fluorescent dyes (e.g., carboxyfluorescein succinimidyl ester (CFSE) or Oregon Green) as well as proliferation markers like 5-bromo-2′-deoxyuridine (BrDU) or Ki67, along with the detection of cytokines provides better specificity for cell identification and allows for detailed phenotyping [18,30,32]. However, these alternatives offer a lower sensitivity and make them less suitable for the identification of small populations of proliferating cells.

Various adjustments have been implemented to increase assay sensitivity, including modification of T cell and APC populations. Blood monocyte-derived DCs (abbreviated MDDCs or MoDCs) or monocyte-derived Langerhans cells (MoLCs) are commonly used APCs [17,18,91,92]. In the human T cell priming assay (hTCPA), additional stimulation to support effective T cell priming has been used, e.g., the addition of CD28 antibodies for T cell co-stimulation or maturation of immature MoDCs with stimulators like lipopolysaccharide (LPS) [18,70]. However, it is important to note that once MoDCs mature, they exhibit a significant reduced phagocytic capacity, which may limit their ability to effectively take up and process haptenated proteins [92]. Other modifications in experimental protocols, such as the addition of cytokines for improved T cell survival, the depletion of several immune-regulatory cells (e.g., CD56+ and CD25+ cells), and/or blocking of inhibitory checkpoint receptors (e.g., programmed cell death ligand-1 (PD-L1) or cytotoxic T-lymphocyte-associated protein 4 (CTLA4)) may contribute to an improved assay sensitivity [32,70,91].

Despite advancements, human priming assays still face difficulties in the reliable identification of sensitizing chemicals. Multiple restimulations may increase background levels due to uneven proliferation of individual T cell clones in samples without antigen stimulation. Optimization for individual parameters within the complex protocol remains labor-intensive and time consuming with prolonged cultivation periods, thus challenging the high-throughput implementation of priming assays [32].

### 3.2. Detection of Memory T Cells

Assays that evaluate the secondary immune response mediated by expanded effector or memory T cells usually aim to detect chemical-specific T cells in previously sensitized individuals. Naïve T cells do not typically proliferate in PBMC cultures unless they receive both antigen-specific TCR engagement and co-stimulatory signals along with cytokine support due to insufficient priming stimulation [86,87,88,92]. On the other hand, effector or memory T cells can be efficiently detected. However, in a regulatory context it is crucial to reach the detection limit in non-allergic donors. For some chemicals, like metals, specific T cells can easily be identified in vitro. In contrast, other chemicals are more challenging, e.g., due to low frequencies of specific T cells and/or difficulties in epitope generation (see Section 4.1 and Section 4.4).

#### 3.2.1. Lymphocyte Proliferation Test (LPT)

The lymphocyte proliferation test (LPT), also termed lymphocyte transformation test (LTT) or lymphocyte stimulation test (LST), measures the antigen-specific response of effector or memory T cell populations based on their in vitro proliferation [16,74,93,94,95]. It is also referred to as a lymphocyte activation test (LAT) in some literature [96,97]. PBMCs, including both T cells and mainly monocytes and B cells as APCs, are cultured with the chemical of interest (or otherwise chemical-induced epitopes, see Section 4.1), usually using replicates. Cell proliferation is measured after several days, typically by [^3^H]-thymidine incorporation and subsequent determination of the stimulation index (see Section 3.1). Alternative readouts, like cytokine production or the expression of several activation markers, can also be combined with the proliferation protocol [98,99]. The addition of several cytokines in cell culture medium has been proposed to enhance assay sensitivity, but their use remains controversial as it may skew overall T cell responses by selectively activating or suppressing specific T cell subsets [99,100].

One main challenge of the LPT is that it does not provide information on original specific cell frequencies unless performed in the setting of limiting dilution cultures. In addition, the results of the LPT may reflect altered frequencies of T cell subpopulations due to varying division speeds [101].

#### 3.2.2. Cytokine-Based Tests

Cytokine-based assays aim to quantify cytokine production by TCR-activated effector or memory T cells and are commonly used as an additional readout of LPT or priming assays [18,77,78].

Among these methods, the enzyme-linked immunosorbent assay (ELISA) or bead-based immunoassays are widely employed to measure cytokine concentrations in cell supernatants. Depending on the applied method, a single cytokine (ELISA), or a variety of soluble analytes (e.g., cytometric bead array (CBA)) can be detected at a time [72]. However, supernatant analysis does not identify cytokine production at single-cell level. In contrast, ELISpot assays visualize cytokine-releasing cells as colored spots, enabling the quantification of responsive individual T cells, though without preserving cell viability [78,102]. In most studies, IFN-γ and IL-10 are the main redout, although ELISpot enables the detection of a variety of cytokines in parallel, e.g., IL-2, IL-4, IL-5 or IL-13, to improve qualification of the induced response [103]. Capture assays, such as the cytokine secretion assay (CSA), also termed cytokine catch assay, detect cytokines at individual cell level while maintaining cell viability [80]. Another approach is intracellular cytokine staining (ICS) using multiparameter flow cytometry. This method employs protein transport inhibitors to prevent extracellular cytokine migration, followed by cell fixation and permeabilization to allow the penetration of fluorochrome-conjugated antibodies. While ICS enables analysis of multiple cytokines and the phenotyping of responsive cells, it does not preserve cell viability [79,104]

The isolated quantification of cytokines as a single readout is associated with certain limitations. The complex nature of cytokine production makes it difficult to efficiently capture antigen-specific responses at any given time point, as not all chemical-specific T cells produce all cytokines [105]. Additionally, elevated or altered baseline cytokine levels, especially during active inflammatory processes, can affect assay interpretation. Furthermore, the detection of T cell subsets with low cytokine profiles can be challenging, leading to incomplete characterization of certain T cell responses [106].

### 3.3. Detection of Both Naïve and Memory T Cells

#### 3.3.1. Peptide-Major Histocompatibility Complex Class I and II Oligomers

The peptide-MHC oligomer technique analyzes the binding of T cells to specific antigens via the interaction between TCRs and recombinantly generated soluble peptide-MHC complexes. Fluorescent tetramers, as well as dimers, pentamers, and higher-order oligomers of MHC I and II molecules loaded with specific peptides, bind to CD8+ or CD4+ T cells, respectively. Antigen-specific T cells are then identified using multiparameter flow cytometry, which also allows for T cell phenotyping and isolation of specific viable T cells [82,107].

The multimer approach is necessary to overcome the low avidity of TCRs, particularly for CD4+ T cells, and stabilize their interaction with MHC [108,109,110]. However, it remains unclear whether MHC oligomers can efficiently capture all relevant T cells, regardless of their activation state or epitope avidity [111,112]. Nevertheless, the adaptability of the assay for detecting chemical-specific T cells has been demonstrated by the development of a nickel mimotope tetramer [107].

Optimized protocols with magnetic-bead enrichment improved assay detection capabilities, allowing for the analysis of rare antigen-specific T cells even in unexposed individuals [110,112]. Besides, a combination with mass cytometry is possible. In this approach, antibodies labeled with isotopically purified metal conjugates are measured by a time-of-flight mass spectrometer. This offers a wider range of parameter detection than flow cytometry and fewer variations in signal intensity between parameters [113,114].

The main bottleneck of the peptide-MHC oligomer technique is that all relevant contributors need to be known, including the presented peptide, the presenting MHC protein and, in the case of chemical sensitizers, the haptenated entity. Moreover, the complex production of peptide-MHC tetramer complexes, especially for MHC II, restricts its widespread use [113,115,116].

#### 3.3.2. Amplified T Cell Libraries

The in vitro generation of amplified T cell libraries enables the quantitative and functional analysis of naïve and memory antigen-specific CD4+ and CD8+ T cells [83]. Initially, T cells are isolated based on their naïve or memory phenotype, followed by polyclonal expansion and subsequent stimulation of fractions of the expanded pools with autologous monocytes, in the presence of an antigen. T cell proliferation of expanded antigen-specific T cell clones is assessed via [^3^H]-thymidine incorporation or flow cytometry and can be combined with further TCR repertoire analysis and cytokine detection assays [83,117,118].

This method offers high sensitivity in the detection of rare antigen-specific T cells. However, it involves a labor-intensive process with extended culture times that requires advanced expertise. Additionally, the in vitro expansion of T cells may not accurately reflect in vivo scenarios due to possible outgrowth or loss of certain specific clones. Moreover, potential phenotypic or functional alterations in T cells induced by in vitro manipulation may also limit reproducibility of real-life settings [40,117]. To date, this method has not been applied to chemical allergens.

#### 3.3.3. Autologous Skin Explant Test

The human in vitro skin explant assay presents an alternative approach to assess the skin sensitization potential of chemicals through a co-culture of MoDCs and T cells from healthy individuals combined with a skin explant from the same donor [84,85,119]. Not unlike priming assay, fast MoDCs are generated (day 1), matured with maturation stimuli (day 2), modified with chemical allergens (day 3), and then co-cultured with T cells (days 4–7). Here, a comprehensive T cell pool is used (CD14- PBMC fraction), which include all T cell subpopulations. Thus, the T cell responses observed in the assay may consist of either primed naïve T cells or (cross-reactive) memory responses and includes CD4+ as well as CD8+ T cells. Then, skin biopsies are co-cultured for another 3 days (days 8–10).

By including a skin explant besides dendritic cell models and T cells, this assay mimics the human in vivo relevant cutaneous immune response more closely compared to other tests. Histopathological damage is the major readout and evaluated according to the Lerner criteria, using hematoxylin and eosin staining [120]. The visible damage in histological sections mainly reflects the response of keratinocytes to the inflammatory environment generated by the in vitro culture, likely dominated by the proliferated, activated chemical-specific T cells. Additional readouts from parallel MoDC-T cell co-cultures include cytokine (IFN-γ) assessment in the cell culture supernatants and T cell proliferation via [^3^H]-thymidine incorporation. The assay has also been applied to biologicals and small-molecule drugs, besides a comparatively large pool of sensitizing chemicals [85,119]. However, the complexity of the in vitro system may also pose some challenges, such as the necessary logistics to obtain autologous skin samples.

#### 3.3.4. Activation-Induced Marker (AIM) Assays

AIM assays offer a short-term functional approach for the detection, isolation, and potential expansion of antigen-specific T cells, even from low original precursor frequencies. These assays are based on the upregulation of surface proteins (activation markers) upon TCR stimulation [40,121,122].

The background expression of the activation markers can complicate the accurate distinction of low-frequency T cell subsets, which compromises assay sensitivity [123,124]. Ideally, these markers should be expressed only after TCR interaction, independently of cytokine secretion or cell differentiation stages, with fast kinetics sustained for a reliable period [40,125]. Some early activation markers like CD69 are intrinsically expressed on several T cell subsets or can be induced by TCR-independent signals [124,126]. Late activation markers, including HLA-DR (HLA-DR) and CD38, show high expression variability and are also prone to TCR-independent bystander activation [127,128,129]. In contrast, CD154 (CD40L) is transiently expressed after 5–7 h on activated CD4+, and to a lesser extent among CD8+ T cells, upon TCR engagement, with minimal bystander activation within this time window [130]. CD154 is rapidly internalized after interacting with CD40, so its detection is only possible intracellularly or with the concomitant addition of a CD40-blocking antibody [106,123,131,132]. Another well-known marker is CD137 (4–1BB), which is upregulated primarily in CD8+ but also CD4+ T cells. Its expression generally peaks around 24 h, with regulatory CD4+ T cells showing peak upregulation at approximately 5 h, and it remains detectable for up to 5 days [125,133,134,135]. Other surface proteins described in the literature include CD134 (OX40) and programmed death-ligand 1 (PD-L1, CD274), which are typically upregulated after 18–24 h [105,133]. It often remains unclear whether the expression of these activation markers varies between cell types or in response to different antigens. The lack of comparative data hinders a comprehensive understanding and the accurate characterization of immune responses, particularly concerning low-frequency T cell subsets [122].

The co-staining of activation markers, e.g., CD154 or CD137 with CD69 and Nur77, an intracellular marker for TCR-mediated activation, can further confirm T cell specificity [40,126,136]. Other markers allow for simultaneous analyses, such as cell proliferation, single-cell cytokine detection, or T cell phenotyping, which also informs on in vivo relevance [19,40,51,52,105]. For instance, HLA-DR, a late activation marker, and Ki-67, a nuclear protein only expressed during active cell division, are often detected among antigen-specific T cells [19,137]. Increased co-expression of cutaneous leukocyte-associated antigen (CLA) on activated cells indicates skin-homing responses [138,139]. However, if the frequency of antigen-specific T cells is low, the analysis of further markers may not be feasible.

Using flow cytometry, only a limited number of cells can be acquired and computationally processed within a reasonable timeframe, which contrasts with the need for a sufficient number of target events for statistically robust analysis, particularly for certain subpopulations [140]. To address these limitations, the antigen-reactive T cell enrichment (ARTE) protocol has been developed as a modification of the AIM assay. This protocol involves magnetic pre-enrichment of activation marker-positive cells, allowing for the acquisition and analysis of rare antigen-specific viable cells from large blood samples [121,140]. Currently, ARTE has successfully been established for CD154 and CD137 [124,141]. Another approach for flow cytometry is to acquire events specifically from target T cell subpopulations to reduce data volume [19,51]. In both options, a small fraction of cells is analyzed in detail to capture original frequencies within parent populations, providing information about cell phenotype and functionality.

Further optimization of AIM assays, e.g., in analogy to priming assays, is possible and it has to be tested whether this leads to improved chemical-specific T cell detection, e.g., using co-stimulation with CD28 or depletion of regulatory T cells [142].

Overall, AIM assays offer promising advantages for both laboratory and clinical applications. Their shorter experimental time leads to fast results and facilitates their large-scale application. Because T cells do not divide during short-term stimulation, T cell frequencies, phenotypes, and TCR profiles obtained remain unbiased, accurately reflecting the in vivo repertoire and frequencies [143]. Specifically, the CD154-based CD4+ AIM assay has proven to be highly effective in the identification of chemical-specific T cells, underscoring its value for monitoring chemical-related immune responses [19,36,52].

## 4. Critical Steps for T Cell Assay Development

**Figure 3:**
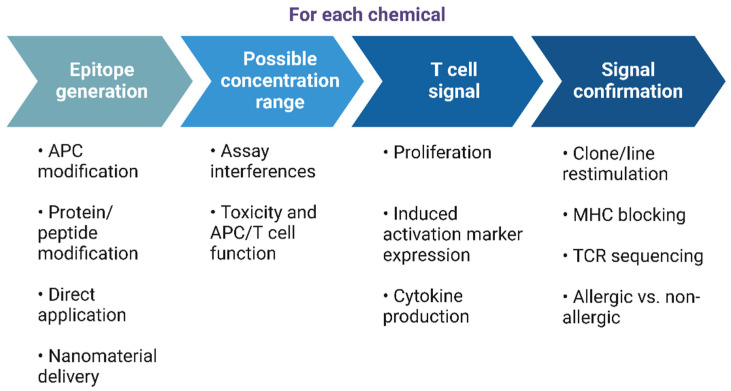
Key steps in the development of in vitro T cell assays. For each key step (large blue arrows), several experimental options can support the analysis of a new chemical (see also Section 4.1, Section 4.2, Section 4.3 and Section 4.4 below). APC—antigen presenting cell; MHC—major histocompatibility complex; TCR—T cell receptor. Created in BioRender.com. Siewert, K. (2024) https://BioRender.com/j18a303, accessed on 1 November 2024.

Several crucial steps are involved in the development of T cell tests (Figure 3), which are discussed in the following sections.

### 4.1. Generation of T Cell Epitopes

For most sensitizers, the identity of the induced T cell epitope and the conditions required for efficient in vitro generation are not well understood. This renders the development of T cell tests for chemical sensitizers more challenging compared to those for protein antigens. The physicochemical properties of the sensitizer drive the molecular mechanisms of its interaction with cellular components. Different aspects need to be considered, including protein binding affinity at different sites, chemical reactivity (including pre- or pro-haptens), or solubility [45,144]. Covalent protein binding (haptenation) can occur on extracellular proteins, which are subsequently processed by APCs, resulting in hapten-derived peptides being presented on MHC II molecules to CD4+ T cells [145,146,147]. Cross-presentation enables the activation of CD8+ T cells due to presentation of haptenized peptides on MHC I molecules. Some haptens can penetrate into the cell and modify intracellular proteins to serve as an epitope source, which is subsequently shuffled into the MHC I presentation pathway [146,147]. Moreover, some chemicals have been shown to covalently modify peptides bound to MHC molecules, leading to the formation of TCR epitopes in a processing-independent way [145,148]. Others may alter intracellular processes to produce neo-epitopes without chemical contact of the TCR [36,149,150]. As a second main mechanism for epitope formation, some chemical-derived epitopes are formed in a processing-independent way [151]. This has been described for metal allergens where epitopes are formed via complex formation (coordination bonds), also termed pharmacological interaction (or p-i) in the context of drug hypersensitivity [36,49,152].

In real life, proteins in the skin are typically modified. Such skin proteins are rarely available in in vitro assays, but this may not pose a problem. According to the concept of T cell polyspecificity, sufficiently similar modified proteins or peptides can be processed and presented by the APCs in the assay, thus engaging a representative fraction of the relevant chemical-specific TCR repertoire, similar to the in vivo skin responses [95,153,154].

**Figure 4:**
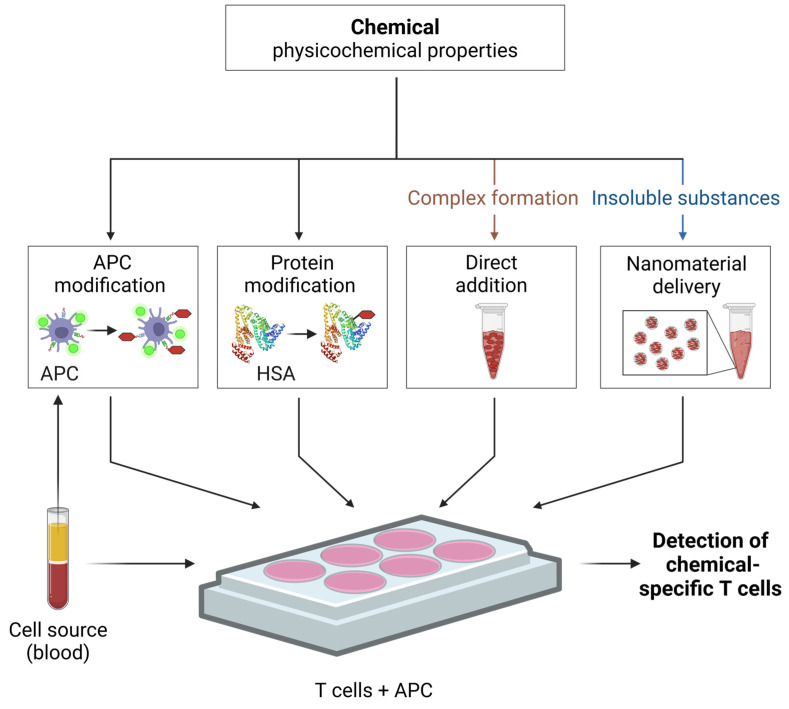
Approaches for the in vitro generation of chemical-induced T cell epitopes. The physicochemical properties of a substance influence which epitope formation approach may be the most suitable. For instance, certain features, like binding via complex formation (chelation) or insolubility, may require direct addition into the culture medium or benefit from nanomaterial-mediated delivery, respectively. Human blood samples serve as the cell source (e.g., for PBMCs, indicated here as white cellular layer after blood density gradient centrifugation), which includes fresh blood donations, cord blood, buffy coats, and leukocyte depletion filters. APC—antigen presenting cells; HSA—human serum albumin. Created in BioRender.com. Siewert, K. (2024). https://BioRender.com/b98e522, accessed on 1 November 2024.

Considering the possible molecular mechanism for epitope formation in vivo, the following four major in vitro approaches can be distinguished in the literature: separate modification of the APCs, modification of model proteins, which are provided to the APCs, direct addition of a chemical to the culture medium, and chemical delivery by nanomaterials (Figure 4).

For sensitizers that interact via covalent protein binding (haptenation), APC modification or the addition of modified proteins (or peptides) to the APCs have been described. APC modification, also termed pulsing, facilitates efficient antigen processing under culture conditions. Additionally, pre-haptens, which require prior oxidation, or pro-haptens, which require metabolic activation, can convert into their immunogenic reactive forms. In contrast, a second way comprises a short-term incubation with a chemical for ~10 min in PBS which may limit toxic effects due to the shorter exposure time. This latter approach allows for a targeted modification of pre-existing surface peptide-MHC complexes, due to the lack of reactive proteins in the cell culture medium [52,70]. To determine if a chemical-induced epitope requires antigen processing, APCs can be fixed with glutaraldehyde, which leads to an immediate stop of antigen uptake and processing [18,70].

The use of haptenated model proteins provides an excess source of extracellular protein for APC processing. Human serum albumin (HSA) is often used as a model protein due to its several lysine and cysteine residues, which readily react with many sensitizers, including nickel and 2,4-dinitrobenzenesulfonic acid [70,155]. Moreover, HSA is the most abundant plasma protein and a common carrier protein in the skin [156,157]. The modification of a single protein provides a limited number of functional groups and consequently, a restricted range of potential T cell epitopes [153,158]. To better mimic the extensive protein diversity encountered during skin sensitization, the modification of a well-defined mix of model proteins (e.g., skin) may improve in vitro epitope formation [159]. Each haptenated model protein or peptide should also be tested without chemical modification to determine background T cell reactivity. Successful protein modifications can be determined analytically, e.g., by mass spectrometry.

The direct addition of the chemicals to the cell culture medium is a straightforward approach to induce T cell responses but requires careful controls for toxicity and cell functions (see Section 4.2). For chemicals that form T cell epitopes via complex formation, such a direct application is essential to maintain the reaction equilibrium.

As an additional approach, the encapsulation within nanomaterials potentially offers an improved epitope formation for insoluble chemicals by maintaining availability, dispersibility and stability in aqueous solutions. Nanomaterials may be taken up by the APC and deliver chemicals for intracellular protein modification. This approach potentially boosts T cell activation, further preventing chemical aggregation and protecting against metabolic or oxidative degradation. It also enables the controlled release of haptens over time, thus mimicking in vivo conditions [160,161,162]. As the use of nanomaterials is already a dynamic field in drug delivery research, an adaptation to the assessment of chemical sensitizers in T cell tests seems promising.

### 4.2. Determination of Suitable In Vitro Assay Conditions

For each chemical and epitope generation approach, suitable chemical concentration ranges should be determined by performing a concentration-dependent stimulation to assess assay interferences and potential toxic effects.

Autofluorescence, which is an inherent property of several chemicals, can complicate the detection of fluorescence-based parameters. To address this issue, it is possible to either select autofluorescence-free flow cytometry channels or to employ alternative detection methods, such as cytometry by time-of-flight (CyTOF) or through [^3^H]-thymidine incorporation. Moreover, chemical binding to the cell membrane can interfere with the binding of viability stains or antibodies, thereby affecting flow cytometry results (unpublished own observation) [163,164]. However, assay interferences due to reduced antibody binding are unlikely to occur when modified APCs or proteins are used as the bases of epitope formation.

Monitoring cell viability and functionality is essential for the accuracy and reliability of T cell assays. Multiparameter flow cytometry is widely used for this purpose, as it allows for the discrimination between viable, apoptotic, and necrotic cells across different cell types and subpopulations [165]. Depending on the kind of epitope formation approach, monitoring the functionality of the T cells and/or the APCs, often a more sensitive parameter than cell viability, is necessary. Proper cell function can be assessed by concurrent incubation of a positive control with the chemical concentrations of interest. The positive control should trigger widespread activation in a processing-independent manner to determine if T cell function is affected. A decrease in control-mediated T cell activation in response to increasing chemical concentrations will indicate functional defects. For this purpose, polyclonal stimulation controls, such as the superantigen staphylococcal enterotoxin B (SEB), have been employed recently in AIM assays [51,52]. It is also imperative to monitor the viability and function of APCs, particularly with regard to the processing-dependent formation of epitopes. Co-exposure to a universal, processing-dependent antigen may help to evaluate APCs’ function (Seifert et al., in preparation). Of note, a preserved APC function during the initial hours of antigen incubation can be sufficiently effective to generate chemical-induced epitopes and specific T cell activation. In contrast, chemicals that act as pre- or pro-haptens may require functional APCs for extended periods.

If solvents like dimethyl sulfoxide (DMSO) are used, their effects on cell viability and function should be carefully monitored. To exclude solvent-related effects, a consistent concentration of the solvent should be maintained throughout the experimental protocol [166,167].

The tolerable concentration of a chemical in the assay can differ depending on the incubation time and the type of cells used. For example, PBMCs in AIM assays may tolerate higher chemical concentrations compared to PBMCs in proliferation assays, due to the shorter experimental time. Careful adjustment of all the described parameters is essential to ensure that assay conditions accurately reflect biological responses. Accordingly, the concentration selected for the T cell assay should be the highest non-toxic level, for example preserving 75–80% of the signal detected in the assay readout. Subsequently, lower concentrations are expected to have no toxic effects. These adjustments will guarantee the reliability and reproducibility of assays while minimizing potential confounding effects on T cell function and assay outcomes.

### 4.3. Confirmation of Chemical-Specific T Cell Responses

Once a signal is obtained from a T cell test, it should be verified to distinguish between true TCR-mediated responses and bystander activation. For instance, higher nickel concentrations were previously interpreted as nonspecific mitogenic effects [168,169]. However, AIM assay data confirmed that nickel-specific T cell responses are induced via the interaction of Ni^2+^ ions with certain features of the TCR repertoire [19,51].

In most assays, the confirmation of T cell signals has traditionally relied on the generation of clones or lines from activated cells, followed by restimulation with the original antigen. Since all T cell clones originate from a single T cell, they share the same TCR sequence and antigen specificity. If these clones react positively upon re-exposure to the original antigen, it confirms specific TCR involvement. These clones can be further characterized for research purposes through cytokine and chemokine analysis or HLA typing. In addition, this method allows for the assessment of potential cross-reactivities by demonstrating positive responses to non-original but related antigens [40,51]. However, the experimental protocol for generating clones is labor-intensive and time-consuming, involving several steps such as culture with feeder cells, expansion, and splitting if necessary. Furthermore, clone restimulation requires autologous APCs, like CD3-depleted PBMCs or Epstein–Barr virus (EBV)-transformed B cells. This can be challenging due to the limited availability of blood samples or the biosafety level 2 requirements, respectively [40,121,139].

To verify MHC restrictions, MHC-blocking antibodies or isotype controls can be employed. These antibodies prevent T cell activation by targeting specific MHC molecules, whether class II (HLA-DR, -DQ or DP) or class I (HLA-A, -B and -C) [40,51,52,170]. This approach is most effective with T cell clones or lines, because blocking antibodies may not be completely effective, particularly due to differences in HLA alleles. As a result, small signals, such as those frequently obtained in assays using bulk PBMCs, may be less reliably controlled by MHC blocking [40,51,52].

Distinctive TCR repertoire features or CDR3 motifs can also serve as indicators of chemical specificity in activated T cells. For example, in nickel-specific T cells, an overrepresentation of the TRAV9-2 gene segments (TAV9-2) and histidine in the CDR3 region indicates TCR-mediated activation [19]. Similar results have been observed for cobalt and palladium and the experimental allergen TNBS, but these features remain to be determined for other chemicals [51,52].

Lastly, differences in cytokine production, phenotypes, proliferation, or frequencies of specific T cells between allergic and non-allergic individuals can indicate specific T cell activation. Increased signals in allergic individuals also demonstrate that the in vitro test accurately reflects in vivo immune responses, as has been shown for nickel or p-phenylenediamine (PPD) [19,171].

### 4.4. T Cell Test as Diagnostic Tool

The gold standard for diagnosing ACD in clinical practice is the patch test which, despite its widespread use, has several limitations. The patch test is an epicutaneous provocation test in which the allergic skin responses of a patient to small amounts of potential allergens on the back are evaluated at repeated time points. One difficulty is that patients with active skin conditions may be unable to undergo testing. In addition, there is a lack of test preparations for less common allergens, which are not included in the predefined patch test panel [172,173]. Furthermore, some allergens may not penetrate the skin efficiently. This phenomenon has been observed in studies examining the reactivity of palladium, a metal allergen, and in investigations of tattoo ink reactions [174,175].

Blood-based in vitro T cell tests, particularly LPT and AIM assays, provide several advantages over the current diagnostic standard [99,176]. They eliminate the discomfort associated with patch testing and reduce the need for multiple visits in the clinic. Particularly, AIM assays offer short processing times, which enables the evaluation of the allergic state within ~48 h after blood sampling. Additionally, in vitro readouts provide a less subjective result compared to patch tests readings and a wider range of potential allergens beyond those applicable in patch test preparations can be tested. Since in vitro tests do not rely on skin penetration, systemic reactions, like adverse drug reactions or implant failure due to allergic reactions, may be more readily detectable with a blood-based assay. Documented half-lives for antigen-specific memory T cells, as seen with vaccinations or infections, and general clonal persistence in longitudinal studies indicate that increased T cell frequencies can be detected over time [42,177,178,179]. In addition, acute eczema and the related ongoing T cell responses may be more clearly identified and linked to the causative allergen. For example, patients who recently underwent patch tests exhibited a marked increase in nickel-specific CD154+CD4+ memory T cells that were also frequently CLA-positive, likely associated with active skin eczema, compared to patients tested in the distant past [19,180]. Thus, in vitro T cell tests would offer an accurate, comprehensive, and more patient-friendly diagnosis of contact allergies. In addition, improved allergy diagnosis can also provide data to further improve regulatory risk assessment.

Still, current challenges limit the replacement of patch tests by in vitro tests, which are not yet accepted as diagnostic tools [181]. One key issue is how well blood reflects the immune status of the skin, given that some T cells circulate between the two compartments while others remain resident [43,182]. Other limitations include the need to better understand and optimize the in vitro epitope generation and the selection of appropriate concentrations for each chemical (see Section 4.1 and Section 4.2). Particularly, the CD154- and CD137-based AIM assays for antigen-specific CD4+ and CD8+ T cell detection have potential for further development as a diagnostic tool [19,125,140]. However, studies comparing current clinical diagnostic methods, including patch testing, medical history, and relevance assessment, with T cell test results are needed.

In general, positive results from diagnostic tests indicate sensitization, whereas the actual manifestation of allergic symptoms, referred to as in vivo relevance, may vary. Consequently, a positive patch test or induced T cell activation may not always accurately reflect in vivo conditions. In vitro, excessively high chemical concentrations may lead to extended T cell activation that may not reflect real-life scenarios, as described for palladium [51,174]. Therefore, determining the range of local in vivo concentrations of chemicals in the skin and/or draining lymph nodes, known as exposure assessment in risk assessment, would be crucial to interpret T cell test results. This topic, however, lies beyond the scope of this review.

## 5. Remaining Challenges and Future Directions

The regulatory implementation of predictive T cell tests for sensitizing chemicals requires a strategic approach that overcomes several challenges. Ideally, chemical-specific T cells will be detected using PBMCs from blood donations and thus likely non-allergic individuals, which requires clarification of ethical issues. The number of required blood samples that is needed to cover both intra- and inter-donor variations, arising from, e.g., sampling effects, differences in TCR repertoires, HLA haplotypes, or immune status and age, still needs to be determined. If rarer alleles are involved, a test with a limited number of donors will not reliably detect T cell responses associated with specific HLA haplotypes. While no HLA associations have been identified for contact allergies, certain drug hypersensitivities are linked to specific HLA haplotypes [47].

Another relevant question is whether to interrogate the naïve or memory T cell pool. The naïve T cell pool is more diverse, but the memory pool may still contain a sufficiently extensive TCR repertoire to detect chemical-specific T cells [19,26,45]. Memory T cells are easier to handle, as they do not require priming. They have already been preselected for antigen reactivity, which means they potentially comprise a higher proportion of responsive TCRs. Pre-existing memory T cells may even be recruited during chemical sensitization in vivo, as shown analogously for memory B cells [183]. However, it is difficult to trace their specific involvement because TCRs do not undergo somatic hypermutation like B cells. Moreover, memory T cells have already undergone differentiation and may not fully translate innate signals from chemical-treated APCs into phenotypic changes as effectively as naïve T cells when used in priming assays.

The assessment of T cell cross-reactivity to predict potential cross-allergies is a major unmet regulatory need. Currently, the most commonly used method involves the generation, expansion, and restimulation of chemical-specific T cell clones or lines, which is highly labor-intensive. In silico methods are not yet capable of predicting TCR reactivity [184]. Recently, bulk high-throughput TCR sequencing has emerged as a promising solution to assess cross-reactivity among activated expanded memory T cell clonotypes identified by the AIM assay. Cross-reactivity must be analyzed within the T cell pool from the same individual, as TCR repertoires are unique to each person. The assessment of the *β*-chain is sufficient to identify cross-reactive clonotypes, but additional α-chain analysis may reveal unique binding sites [19,51].

A T cell assay could offer valuable insights in a tiered approach for hazard identification, particularly in the assessment of chemicals that show discrepancies or unclear results in current KE1-3 NAM, LLNA, and human data. However, a negative T cell test result remains inconclusive until more experience is gained with known skin sensitizing chemicals. In this context, in vitro T cell assays may not always provide sufficient predictions of human clinical outcomes but rather complement existing approaches. T cell tests alone may underestimate severe adverse reactions, if they do not effectively mimic in vivo conditions for antigen presentation. This emphasizes the need for more comprehensive and integrated approaches for safety assessment beyond in vitro T cells tests alone. As a final objective, the practical application of in vitro T cell assays within regulatory frameworks would be highly beneficial. Currently, the state of T cell tests for regulatory purposes could be assigned a regulatory readiness level (RLL) of 2 (mid research stage), as reviewed and outlined by Haase et al. [185] The scientific relevance should be further illustrated by case studies. Standardization and validation of these assays must follow a structured pathway to ensure their acceptance and implementation. First, internal validation must be achieved with a clear protocol (SOP; standard operating procedure) with a well-defined endpoint and applicability domain [32,185]. This preliminary process also includes the validation of the accuracy and reproducibility of the assay. Next, large-scale inter-laboratory validation studies should be conducted to further confirm the transferability, reproducibility, and reliability of the assays. In these trials, participating laboratories will adhere to the established protocol while conducting independent assays with their own setups, to assess the effectiveness of the assays across diverse environments. To the best of our knowledge, there is currently no established standardization or inter-laboratory collaboration specific to a regulatory T cell test. However, upon successful completion and data analysis to determine accuracy, predictive capacity, applicability domain, and performance standards of an assay under these settings, a final report can be submitted to a recognized validation organism, such as the European Union Reference Laboratory for Alternatives to Animal Testing (EURL ECVAM), which will evaluate whether the method meets the necessary criteria for formal validation. After successful validation and potential approval, the assays can be proposed for inclusion in the OECD test guidelines, facilitating their acceptance in regulatory frameworks throughout the EU and other OECD member countries [186,187]. Nevertheless, since not all NAMs can be prioritized for inclusion in the OECD test guidelines, a potential alternative which is currently in evaluation is the establishment of a qualification system. This system is assessed by experts to ensure that NAMs are used effectively in suitable contexts and to help optimize them for regulatory applications [185]. Besides, continuous monitoring of the assay protocol is essential to maintain ongoing effectiveness and relevance. Looking ahead, it will be important also to assess respiratory sensitizers and drugs, including biologicals [85,119]. Further, NAMs should aim to replace or reduce the use of animal-derived products, such as bovine serum or animal-derived antibodies, with several options already available [188,189].

An important emerging field comprises efforts towards a precise assessment of the potency of skin sensitizers, i.e., the dose per cm^2^ of skin associated with a certain degree of sensitization within a fraction of individuals, optimally measured on a continuous scale [190,191,192]. This information is relevant as it should help to define safe levels and enable risk assessment beyond hazard identification. Potency data are derived from dose-response curves (hazard characterization). A combination of methods from the different KEs in the skin sensitization AOP is likely needed to obtain reliable values. Integrating findings from various testing methods, such as T cell assays, could lead to better-defined applicability domains for chemicals, ultimately enhancing regulatory risk assessment. It is still unknown to which extent immunological responses, which may include tolerance and threshold mechanisms, follow conventional dose-response curves [193,194]. Concentration-dependent T cell activation has been shown for metal allergens binding via complex formation [19,51]. However, how and whether frequencies of in vitro reactive T cells can be translated into potency data is still uncertain and further experience with additional chemicals is needed [26,31,32].

## 6. Conclusions

Overall, T cell assays remain demanding experimental methods due to the intricacies of chemical-induced epitope generation, combined with the need to identify specific T cells among numerous irrelevant bystanders. Recently, AIM assays have been adapted to investigate skin sensitizers [19,51,52]. Particularly, CD154-based AIM assays are fast, highly sensitive, comprehensive, and directly quantitative methods for antigen-specific CD4+ T cell responses, supporting an efficient optimization of experimental conditions without prior knowledge of epitopes or cytokine secretion patterns. When combined with bulk TCR high-throughput sequencing, AIM assays can assess T cell cross-reactivity more efficiently than restimulation of specific T cell clones or lines [51].

The in vitro T cell tests presented here highlight the advantages and potential of NAMs by being directly relevant to human conditions, offering ethical benefits, and providing new insights not available from animal models. However, more resources are needed to test more chemicals and facilitate regulatory implementation. While not all chemicals or substances may require T cell testing, bridging the T cell-related gap in NAM-based sensitizing chemical hazard identification could significantly complement and enhance regulatory frameworks.

## Figures and Tables

**Table 1:** In vitro assays used for the identification of human chemical-specific T cells. The remark column mentions general features and example references. In addition, variations, e.g., additional readouts or key experimental details are listed from individual exemplary publications.

Assay	Main Read-Out	Remarks and Example References	Limitations	Example Chemicals
**Naïve T cells**				
Priming assays	proliferation	phenotyping is possible (CD4/CD8)	requires professional APCs to overcome activation threshold of naïve T cells, time-intensive	BB, DNCB, DNBS, Eugenol, FITC, HCA, Isoeugenol, PPD, TNBS
		APCs: cultured LCs [17]		
		APCs: MoDCs [68]		
● hTCPA		depletion of CD25+ regulatory T cells [69]		
		cytokine detection (IFN-γ, TNF-α) [18,70]		
		clone characterization [71]		
				
**Memory T cells**				
LPT	proliferation	cytokines (inverse relation of IL-5 and IL-8) [72]	radioactivity is the most sensitive read-out but prevents down-stream analysis [73,74]	Mercury, Nickel
		phenotyping is possible (CD4/CD8) [75]		
Cytokine-based assays	production of cytokines	frequently analyzed cytokines: IFN-γ, IL-10	not all specific T cells produce a given cytokine	BB, Nickel, PPD
● ELISA ● Bead assays	in supernatant	single cytokines (ELISA) or multiple soluble analytes (multiplex-bead assays) [76,77]	no resolution at single-cell level	
● ELISpot		cytokine detection on single-cell level [78], quantitative	prevents down-stream analysis, no phenotyping possible	
● ICS	cell-based	Parallel analysis of multiple cytokines and phenotyping of responsive cells on single cell level, quantitative	fixation and permeabilization required, thus preventing down-stream analysis of living cells [79]	
● Capture assays		viable single cell analysis enables down-stream analysis [80]	limited cytokine analysis [81]	
				
**Naïve and memory T cells**				
pMHC oligomers		phenotyping and downstream analysis of viable, antigen-specific cells [82]	epitope knowledge required for (chemically modified) peptide-MHC, oligomer production	Nickel (mimotope)
Amplified T cells libraries	proliferation	identification of low frequency clones, HTS possible [83]	work- and time intensive, amplification of single clones may deviate from real-life conditions	
Autologous skin explant test	histo-pathological analysis of human skin	histopathological score for damage induced in skin explants added to chemical-treated MoDCs—T cell co-cultures [84,85]	time intensive, limited availability of skin samples	Cinnamic alcohol, oxazolone
AIM assay	activation- induced surface protein expression	fast, sensitive, quantitative, downstream analysis of life specific T cells (further phenotyping, TCR repertoire analysis) [19,51,52]	less established for antigen-specific CD8 T cells, fluorescence interferences may limit analysis of high chemical concentrations	TNBS, Nickel

AIM—activation-induced marker; APC—antigen presenting cell; BB—Brandowsky’s base; CD—cluster of differentiation; DNBS—2,4-dinitrobenzenesulfonic acid; DNCB—2,4-dinitrochlorobenzene; ELISA—enzyme-linked immunosorbent assay; ELISpot—enzyme-linked immunoSpot assay; FITC—fluorescein isothiocyanate; HCA—α-hexylcinnamaldehyde; hTCPA—human T cell priming assay; HTS—high-throughput sequencing; ICS—intracellular cytokine staining; IFN—interferon; IL—interleukin; LC—Langerhans cells; LPT—lymphocyte proliferation test; MoDCs—monocyte-derived dendritic cells; pMHC—peptide major histocompatibility complex; PPD—p-phenylenediamine; TCR—T cell receptor; TNBS—2,4,6-trinitrobenzenesulfonic acid; TNF—tumor necrosis factor.

## Data Availability

No new data were created or analyzed in this study. Data sharing is not applicable to this article.

## Abbreviations

ACD—allergic contact dermatitis; AIM—activation-induced marker; AOP—adverse outcome pathway; APC—antigen presenting cell; CD—cluster of differentiation; DC—dendritic cell; KE—key event; LPT—lymphocyte proliferation test; MHC—major histocompatibility complex; NAM—new approach methodology; OECD—Organization for Economic Co-operation and Development; TCR—T cell receptor; TG—test guideline; TNBS—2,4,6-trinitrobenzenesulfonic acid.
